# *N*-(3-Chloro­benzo­yl)-3-nitro­benzene­sulfonamide

**DOI:** 10.1107/S1600536811054857

**Published:** 2011-12-23

**Authors:** P. A. Suchetan, Sabine Foro, B. Thimme Gowda

**Affiliations:** a Department of Chemistry, Mangalore University, Mangalagangotri 574 199, Mangalore, India; b Institute of Materials Science, Darmstadt University of Technology, Petersenstrasse 23, D-64287 Darmstadt, Germany

## Abstract

In the title compound, C_13_H_9_ClN_2_O_5_S, the dihedral angle between the two benzene rings is 83.5 (1)°. In the crystal, mol­ecules are linked *via* N—H⋯O(S) hydrogen bonds into helical chains running along the *b* axis.

## Related literature

For our studies on the effects of substituents on the structures and other aspects of *N*-(ar­yl)-amides, see: Bowes *et al.* (2003 ▶); Gowda *et al.* (2004 ▶), on *N*-(ar­yl)-methane­sulfonamides, see: Jayalakshmi & Gowda (2004 ▶), on*N*-(ar­yl)-aryl­sulfonamides, see: Gowda *et al.* (2003 ▶), on *N*-(substitutedbenzo­yl)-aryl­sulfonamides, see: Suchetan *et al.* (2011 ▶) and on *N*-chloro­aryl­amides, see: Gowda & Mahadevappa (1983 ▶).
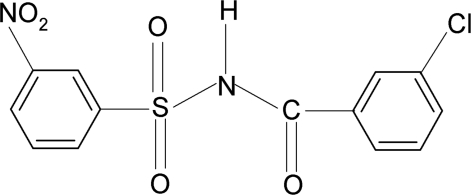


## Experimental

### 

#### Crystal data

C_13_H_9_ClN_2_O_5_S
*M* *_r_* = 340.73
Monoclinic, 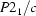

*a* = 11.891 (2) Å
*b* = 5.0577 (6) Å
*c* = 23.488 (3) Å
β = 90.43 (1)°
*V* = 1412.6 (3) Å^3^
*Z* = 4
Mo *K*α radiation
μ = 0.44 mm^−1^
*T* = 293 K
0.46 × 0.20 × 0.10 mm

#### Data collection

Oxford Diffraction Xcalibur diffractometer with a Sapphire CCD detector
Absorption correction: multi-scan (*CrysAlis RED*; Oxford Diffraction, 2009 ▶) *T*_min_ = 0.822, *T*_max_ = 0.957
4873 measured reflections
2840 independent reflections
2204 reflections with *I* > 2σ(*I*)
*R*_int_ = 0.017

#### Refinement

*R*[*F* ^2^ > 2σ(*F* ^2^)] = 0.058
*wR*(*F* ^2^) = 0.105
*S* = 1.20
2840 reflections
202 parameters
1 restraint
H atoms treated by a mixture of independent and constrained refinement
Δρ_max_ = 0.35 e Å^−3^
Δρ_min_ = −0.35 e Å^−3^

### 

Data collection: *CrysAlis CCD* (Oxford Diffraction, 2009 ▶); cell refinement: *CrysAlis CCD*; data reduction: *CrysAlis RED* (Oxford Diffraction, 2009 ▶); program(s) used to solve structure: *SHELXS97* (Sheldrick, 2008 ▶); program(s) used to refine structure: *SHELXL97* (Sheldrick, 2008 ▶); molecular graphics: *PLATON* (Spek, 2009 ▶); software used to prepare material for publication: *SHELXL97*.

## Supplementary Material

Crystal structure: contains datablock(s) I, global. DOI: 10.1107/S1600536811054857/bt5758sup1.cif

Structure factors: contains datablock(s) I. DOI: 10.1107/S1600536811054857/bt5758Isup2.hkl

Supplementary material file. DOI: 10.1107/S1600536811054857/bt5758Isup3.cml

Additional supplementary materials:  crystallographic information; 3D view; checkCIF report

## Figures and Tables

**Table 1:** Hydrogen-bond geometry (Å, °)

*D*—H⋯*A*	*D*—H	H⋯*A*	*D*⋯*A*	*D*—H⋯*A*
N1—H1*N*⋯O2^i^	0.82 (2)	2.29 (2)	3.100 (3)	169 (3)

Symmetry code: (i) 
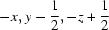
.

**Fig. 1.**
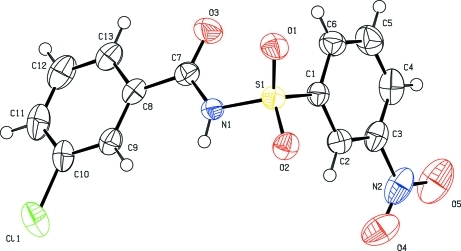
Molecular structure of the title compound, showing the atom-labelling scheme. Displacement ellipsoids are drawn at the 50% probability level.

**Fig. 2.**
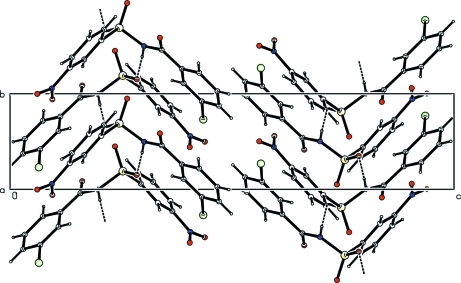
Molecular packing in the title compound. Hydrogen bonds are shown as dashed lines.

## supplementary crystallographic information

### Comment

Diaryl acylsulfonamides are known as potent antitumor agents against a broad spectrum of human tumor xenografts in nude mice. As part of our studies on the substituent effects on the structures and other aspects of *N*-(aryl)-amides (Bowes *et al.*, 2003; Gowda *et al.*, 2004), *N*-(aryl)-methanesulfonamides (Jayalakshmi & Gowda, 2004), *N*-(aryl)-arylsulfonamides (Gowda *et al.*, 2003); *N*-(substitutedbenzoyl)-arylsulfonamides (Suchetan *et al.*, 2011) and *N*-chloro-arylsulfonamides (Gowda & Mahadevappa, 1983), in the present work, the crystal structure of *N*-(3-chlorobenzoyl)-3-nitrobenzenesulfonamide (I) has been determined (Fig.1).

The conformation between the N—H and C=O bonds in the C—SO_2_—NH—C(O) segment is *anti* and the N—C bond in the segment has *gauche* torsion with respect to the S═O bonds(Fig.1), similar to that observed in *N*-(benzoyl)-3-nitrobenzenesulfonamide (II)(Suchetan *et al.*, 2011). Further, in (I), the conformation between the N—H bond and the *meta*-nitro group in the sulfonyl benzene ring is *syn*, similar to that observed in (II). But the conformation of the C═O is *anti* to the *meta*-Cl atom in the benzoyl ring.

The molecule is twisted at the S—N bond with the torsional angle of -60.40 (29)°, compared to the value of -62.80 (17)° in (II).

The dihedral angle between the sulfonyl benzene ring and the —SO_2_—NH—C—O segment is 77.0 (1)°, compared to the value of 79.2 (1)° in (II). Furthermore, the dihedral angle between the sulfonyl and the benzoyl benzene rings is 83.5 (1)°, compared to the value of 86.7 (1)° in (II).

The packing of molecules linked by of N—H···O(S) hydrogen bonds(Table 1) is shown in Fig. 2.

### Experimental

The title compound was prepared by refluxing a mixture of 3-chlorobenzoic acid (0.02 mole), 3-nitrobenzenesulfonamide (0.02 mole) and excess phosphorous oxychloride for 3 h on a water bath. The resultant mixture was cooled and poured into crushed ice. The solid, *N*-(3-chlorobenzoyl)-3-nitrobenzenesulfonamide, obtained was filtered, washed thoroughly with water and then dissolved in sodium bicarbonate solution. The compound was later reprecipitated by acidifying the filtered solution with dilute HCl. It was filtered, dried and recrystallized.

Rod like colourless single crystals of the title compound used in X-ray diffraction studies were obtained by slow evaporation of its toluene solution at room temperature.

### Refinement

The H atom of the NH group was located in a difference map and its coordinates were refined with the N—H distance restrained to 0.86 (2) %A. The other H atoms were positioned with idealized geometry using a riding model with C—H = 0.93 Å. All H atoms were refined with isotropic displacement parameters set to 1.2 times of the *U*_eq_ of the parent atom.

### Crystal data

**Table:**

C_13_H_9_ClN_2_O_5_S	*F*(000) = 696
*M**_r_* = 340.73	*D*_x_ = 1.602 Mg m^−^^3^
Monoclinic, *P*2_1_/*c*	Mo *K*α radiation, λ = 0.71073 Å
Hall symbol: -P 2ybc	Cell parameters from 1638 reflections
*a* = 11.891 (2) Å	θ = 2.4–27.9°
*b* = 5.0577 (6) Å	µ = 0.44 mm^−^^1^
*c* = 23.488 (3) Å	*T* = 293 K
β = 90.43 (1)°	Rod, colourless
*V* = 1412.6 (3) Å^3^	0.46 × 0.20 × 0.10 mm
*Z* = 4	

### Data collection

**Table:**

Oxford Diffraction Xcalibur diffractometer with a Sapphire CCD detector	2840 independent reflections
Radiation source: fine-focus sealed tube	2204 reflections with *I* > 2σ(*I*)
graphite	*R*_int_ = 0.017
Rotation method data acquisition using ω scans	θ_max_ = 26.4°, θ_min_ = 2.4°
Absorption correction: multi-scan (*CrysAlis RED*; Oxford Diffraction, 2009)	*h* = −14→14
*T*_min_ = 0.822, *T*_max_ = 0.957	*k* = −3→6
4873 measured reflections	*l* = −14→29

### Refinement

**Table:**

Refinement on *F*^2^	Primary atom site location: structure-invariant direct methods
Least-squares matrix: full	Secondary atom site location: difference Fourier map
*R*[*F*^2^ > 2σ(*F*^2^)] = 0.058	Hydrogen site location: inferred from neighbouring sites
*wR*(*F*^2^) = 0.105	H atoms treated by a mixture of independent and constrained refinement
*S* = 1.20	*w* = 1/[σ^2^(*F*_o_^2^) + (0.0074*P*)^2^ + 2.6391*P*] where *P* = (*F*_o_^2^ + 2*F*_c_^2^)/3
2840 reflections	(Δ/σ)_max_ = 0.005
202 parameters	Δρ_max_ = 0.35 e Å^−^^3^
1 restraint	Δρ_min_ = −0.35 e Å^−^^3^

### Special details

**Table:**

Experimental. Absorption correction: CrysAlis RED (Oxford Diffraction, 2009) Empirical absorption correction using spherical harmonics, implemented in SCALE3 ABSPACK scaling algorithm.
Geometry. All e.s.d.'s (except the e.s.d. in the dihedral angle between two l.s. planes) are estimated using the full covariance matrix. The cell e.s.d.'s are taken into account individually in the estimation of e.s.d.'s in distances, angles and torsion angles; correlations between e.s.d.'s in cell parameters are only used when they are defined by crystal symmetry. An approximate (isotropic) treatment of cell e.s.d.'s is used for estimating e.s.d.'s involving l.s. planes.
Refinement. Refinement of *F*^2^ against ALL reflections. The weighted *R*-factor *wR* and goodness of fit *S* are based on *F*^2^, conventional *R*-factors *R* are based on *F*, with *F* set to zero for negative *F*^2^. The threshold expression of *F*^2^ > σ(*F*^2^) is used only for calculating *R*-factors(gt) *etc*. and is not relevant to the choice of reflections for refinement. *R*-factors based on *F*^2^ are statistically about twice as large as those based on *F*, and *R*- factors based on ALL data will be even larger.

### Fractional atomic coordinates and isotropic or equivalent isotropic displacement parameters (Å^2^)

**Table:**

	*x*	*y*	*z*	*U*_iso_*/*U*_eq_	
Cl1	−0.01505 (9)	0.2312 (2)	0.06480 (5)	0.0673 (3)	
S1	0.16424 (7)	1.19021 (16)	0.25405 (3)	0.0329 (2)	
O1	0.1952 (2)	1.4485 (5)	0.23632 (10)	0.0459 (6)	
O2	0.06284 (18)	1.1510 (5)	0.28538 (9)	0.0426 (6)	
O3	0.3210 (2)	1.0853 (6)	0.16263 (11)	0.0549 (7)	
O4	0.2384 (3)	0.4415 (6)	0.40468 (13)	0.0754 (9)	
O5	0.4054 (3)	0.4980 (8)	0.43876 (14)	0.0927 (12)	
N1	0.1495 (2)	0.9973 (5)	0.19807 (11)	0.0326 (6)	
H1N	0.099 (2)	0.888 (6)	0.2022 (14)	0.039*	
N2	0.3285 (3)	0.5553 (7)	0.40604 (14)	0.0596 (10)	
C1	0.2775 (3)	1.0595 (6)	0.29449 (13)	0.0329 (7)	
C2	0.2570 (3)	0.8591 (7)	0.33282 (14)	0.0369 (8)	
H2	0.1858	0.7855	0.3366	0.044*	
C3	0.3472 (3)	0.7720 (7)	0.36558 (14)	0.0427 (9)	
C4	0.4536 (3)	0.8782 (9)	0.36104 (16)	0.0529 (10)	
H4	0.5124	0.8157	0.3836	0.063*	
C5	0.4710 (3)	1.0782 (9)	0.32241 (17)	0.0540 (10)	
H5	0.5423	1.1519	0.3189	0.065*	
C6	0.3836 (3)	1.1707 (8)	0.28888 (15)	0.0436 (8)	
H6	0.3957	1.3060	0.2628	0.052*	
C7	0.2344 (3)	0.9602 (7)	0.15852 (14)	0.0373 (8)	
C8	0.2142 (3)	0.7600 (7)	0.11301 (13)	0.0374 (8)	
C9	0.1165 (3)	0.6101 (7)	0.10919 (14)	0.0399 (8)	
H9	0.0580	0.6387	0.1345	0.048*	
C10	0.1067 (3)	0.4200 (8)	0.06786 (14)	0.0451 (9)	
C11	0.1915 (4)	0.3747 (9)	0.02952 (15)	0.0571 (11)	
H11	0.1840	0.2445	0.0018	0.069*	
C12	0.2877 (4)	0.5261 (9)	0.03305 (16)	0.0607 (12)	
H12	0.3454	0.4976	0.0072	0.073*	
C13	0.3000 (3)	0.7185 (8)	0.07404 (14)	0.0473 (9)	
H13	0.3651	0.8202	0.0757	0.057*	

### Atomic displacement parameters (Å^2^)

**Table:**

	*U*^11^	*U*^22^	*U*^33^	*U*^12^	*U*^13^	*U*^23^
Cl1	0.0696 (7)	0.0659 (7)	0.0663 (7)	−0.0096 (6)	−0.0136 (5)	−0.0209 (6)
S1	0.0345 (4)	0.0284 (4)	0.0357 (4)	0.0021 (4)	0.0005 (3)	−0.0003 (4)
O1	0.0571 (16)	0.0278 (13)	0.0526 (15)	−0.0010 (11)	−0.0058 (12)	0.0028 (11)
O2	0.0355 (12)	0.0516 (15)	0.0408 (13)	0.0068 (11)	0.0062 (10)	−0.0020 (12)
O3	0.0418 (14)	0.0674 (18)	0.0557 (16)	−0.0170 (14)	0.0096 (12)	−0.0107 (15)
O4	0.093 (2)	0.062 (2)	0.072 (2)	0.0072 (19)	0.0132 (19)	0.0273 (18)
O5	0.104 (3)	0.108 (3)	0.066 (2)	0.040 (2)	−0.0120 (19)	0.031 (2)
N1	0.0338 (15)	0.0324 (15)	0.0318 (14)	−0.0039 (12)	0.0040 (12)	−0.0014 (12)
N2	0.081 (3)	0.058 (2)	0.0409 (18)	0.031 (2)	0.0058 (19)	0.0080 (17)
C1	0.0352 (17)	0.0316 (17)	0.0320 (16)	0.0022 (14)	0.0005 (13)	−0.0031 (15)
C2	0.0380 (18)	0.0339 (19)	0.0388 (18)	0.0029 (15)	0.0026 (14)	−0.0020 (15)
C3	0.054 (2)	0.042 (2)	0.0327 (17)	0.0148 (18)	0.0003 (15)	−0.0013 (16)
C4	0.047 (2)	0.066 (3)	0.045 (2)	0.020 (2)	−0.0072 (17)	−0.012 (2)
C5	0.0344 (19)	0.072 (3)	0.056 (2)	−0.002 (2)	−0.0006 (17)	−0.010 (2)
C6	0.0395 (19)	0.045 (2)	0.046 (2)	−0.0034 (17)	0.0036 (16)	−0.0016 (18)
C7	0.0375 (18)	0.0384 (19)	0.0360 (18)	0.0004 (16)	0.0017 (15)	0.0049 (15)
C8	0.0418 (18)	0.039 (2)	0.0313 (17)	0.0070 (16)	0.0047 (14)	0.0034 (15)
C9	0.0410 (19)	0.047 (2)	0.0314 (17)	0.0049 (17)	0.0038 (14)	−0.0014 (16)
C10	0.057 (2)	0.047 (2)	0.0319 (18)	0.0084 (18)	−0.0062 (16)	−0.0074 (17)
C11	0.077 (3)	0.060 (3)	0.035 (2)	0.014 (2)	−0.0030 (19)	−0.0113 (19)
C12	0.065 (3)	0.076 (3)	0.041 (2)	0.015 (2)	0.018 (2)	−0.005 (2)
C13	0.049 (2)	0.054 (2)	0.0388 (19)	0.0050 (19)	0.0087 (16)	0.0026 (19)

### Geometric parameters (Å, °)

**Table:**

Cl1—C10	1.736 (4)	C4—C5	1.376 (6)
S1—O1	1.420 (2)	C4—H4	0.9300
S1—O2	1.431 (2)	C5—C6	1.381 (5)
S1—N1	1.645 (3)	C5—H5	0.9300
S1—C1	1.770 (3)	C6—H6	0.9300
O3—C7	1.212 (4)	C7—C8	1.490 (5)
O4—N2	1.217 (5)	C8—C9	1.390 (5)
O5—N2	1.225 (4)	C8—C13	1.391 (4)
N1—C7	1.390 (4)	C9—C10	1.371 (5)
N1—H1N	0.823 (18)	C9—H9	0.9300
N2—C3	1.468 (5)	C10—C11	1.376 (5)
C1—C2	1.378 (4)	C11—C12	1.379 (6)
C1—C6	1.388 (4)	C11—H11	0.9300
C2—C3	1.387 (5)	C12—C13	1.376 (5)
C2—H2	0.9300	C12—H12	0.9300
C3—C4	1.380 (5)	C13—H13	0.9300
			
O1—S1—O2	119.97 (15)	C6—C5—H5	119.7
O1—S1—N1	109.70 (15)	C5—C6—C1	119.3 (4)
O2—S1—N1	104.11 (14)	C5—C6—H6	120.4
O1—S1—C1	107.63 (15)	C1—C6—H6	120.4
O2—S1—C1	108.23 (15)	O3—C7—N1	119.8 (3)
N1—S1—C1	106.45 (14)	O3—C7—C8	123.0 (3)
C7—N1—S1	122.8 (2)	N1—C7—C8	117.2 (3)
C7—N1—H1N	121 (2)	C9—C8—C13	119.5 (3)
S1—N1—H1N	112 (2)	C9—C8—C7	123.1 (3)
O4—N2—O5	123.9 (4)	C13—C8—C7	117.4 (3)
O4—N2—C3	118.2 (3)	C10—C9—C8	119.6 (3)
O5—N2—C3	117.8 (4)	C10—C9—H9	120.2
C2—C1—C6	121.7 (3)	C8—C9—H9	120.2
C2—C1—S1	119.2 (2)	C9—C10—C11	121.5 (4)
C6—C1—S1	119.1 (3)	C9—C10—Cl1	118.8 (3)
C1—C2—C3	117.2 (3)	C11—C10—Cl1	119.8 (3)
C1—C2—H2	121.4	C10—C11—C12	118.7 (4)
C3—C2—H2	121.4	C10—C11—H11	120.7
C4—C3—C2	122.6 (3)	C12—C11—H11	120.7
C4—C3—N2	118.9 (3)	C13—C12—C11	121.2 (4)
C2—C3—N2	118.5 (3)	C13—C12—H12	119.4
C5—C4—C3	118.7 (3)	C11—C12—H12	119.4
C5—C4—H4	120.7	C12—C13—C8	119.5 (4)
C3—C4—H4	120.7	C12—C13—H13	120.2
C4—C5—C6	120.6 (4)	C8—C13—H13	120.2
C4—C5—H5	119.7		
			
O1—S1—N1—C7	55.8 (3)	C4—C5—C6—C1	0.0 (6)
O2—S1—N1—C7	−174.6 (3)	C2—C1—C6—C5	0.1 (5)
C1—S1—N1—C7	−60.4 (3)	S1—C1—C6—C5	176.8 (3)
O1—S1—C1—C2	157.3 (3)	S1—N1—C7—O3	−4.7 (5)
O2—S1—C1—C2	26.2 (3)	S1—N1—C7—C8	174.1 (2)
N1—S1—C1—C2	−85.2 (3)	O3—C7—C8—C9	177.9 (3)
O1—S1—C1—C6	−19.5 (3)	N1—C7—C8—C9	−0.8 (5)
O2—S1—C1—C6	−150.5 (3)	O3—C7—C8—C13	0.0 (5)
N1—S1—C1—C6	98.1 (3)	N1—C7—C8—C13	−178.7 (3)
C6—C1—C2—C3	−0.2 (5)	C13—C8—C9—C10	1.3 (5)
S1—C1—C2—C3	−176.9 (2)	C7—C8—C9—C10	−176.5 (3)
C1—C2—C3—C4	0.2 (5)	C8—C9—C10—C11	−0.5 (5)
C1—C2—C3—N2	−178.9 (3)	C8—C9—C10—Cl1	178.8 (3)
O4—N2—C3—C4	−171.0 (4)	C9—C10—C11—C12	−0.3 (6)
O5—N2—C3—C4	8.2 (5)	Cl1—C10—C11—C12	−179.6 (3)
O4—N2—C3—C2	8.1 (5)	C10—C11—C12—C13	0.3 (6)
O5—N2—C3—C2	−172.7 (3)	C11—C12—C13—C8	0.6 (6)
C2—C3—C4—C5	−0.1 (5)	C9—C8—C13—C12	−1.4 (5)
N2—C3—C4—C5	179.0 (3)	C7—C8—C13—C12	176.6 (3)
C3—C4—C5—C6	0.0 (6)		

### Hydrogen-bond geometry (Å, °)

**Table:**

*D*—H···*A*	*D*—H	H···*A*	*D*···*A*	*D*—H···*A*
N1—H1N···O2^i^	0.82 (2)	2.29 (2)	3.100 (3)	169 (3)

Symmetry codes: (i) −*x*, *y*−1/2, −*z*+1/2.
